# Kappa free light chain concentration in serum is reduced after CD20-depletion with ocrelizumab

**DOI:** 10.1186/s42466-025-00419-7

**Published:** 2025-08-22

**Authors:** Franz Felix Konen, Gudrun Mechthild Körner, Martin W. Hümmert, Philipp Sebastian Gehring, Philipp Schwenkenbecher, Konstantin Fritz Jendretzky, Sandra Nay, Nora Möhn, Lea Grote-Levi, Kurt-Wolfram Sühs, Elke Voß, Refik Pul, Torsten Witte, Thomas Skripuletz, Stefan Gingele

**Affiliations:** 1 Department of Neurology, Hannover Medical School, Hannover, Germany; 2 Department of Neurology, Universitätsspital Zürich, Zurich, Switzerland; 3 Neurocenter Barsinghausen, Barsinghausen, Germany; 4 Department of Neurology, University Medicine Essen, Essen, Germany; 5 Center for Translational Neuro- and Behavioral Sciences (C-TNBS), University Medicine Essen, Essen, Germany; 6 Department of Rheumatology & Clinical Immunology, Hannover Medical School, Hannover, Germany

**Keywords:** Multiple sclerosis, Ocrelizumab, Kappa free light chains, Serum, Biomarker

## Abstract

**Background:**

Kappa free light chains (KFLC), a byproduct of immunoglobulin (Ig) synthesis by B-lineage cells, can serve as an indicator for inflammatory activity. In multiple sclerosis (MS), especially the intrathecal KFLC production has gained increasing importance as a biomarker for central nervous system (CNS) inflammation and was included into the proposed 2024 revision of the McDonald criteria. In contrast, studies investigating the significance of KFLC in serum and the effects of disease-modifying therapies (DMT) on KFLC serum concentration in MS are rare. The aim of the present work was to investigate the impact of B cell depletion with ocrelizumab on KFLC concentrations in serum of MS patients and the ability of serum KFLC to monitor disease activity.

**Methods:**

50 MS patients were included in the present study– 38 with the diagnosis of relapsing MS (RMS) and 12 with diagnosis of primary-progressive MS (PPMS) -, who were treated with ocrelizumab for two years. Serum concentrations of albumin, immunoglobulins and KFLC as well as lymphocyte subsets were determined at baseline and after two years.

**Results:**

Serum Ig and KFLC concentrations were found to be significantly lower after two years of ocrelizumab treatment (mean serum concentrations: KFLC: 9.5 mg/l vs. 7.8 mg/l, *p* = 0.0003; IgG: 9 g/l vs. 8 g/l, *p* = 0.0002; IgA: 2 g/l vs. 1.8 g/l, *p* = 0.0010; IgM: 1.8 g/l vs. 0.7 g/l, *p* < 0.0001). Serum KFLC concentration did not correlate with clinical and paraclinical parameters of disease activity.

**Conclusions:**

Treatment with ocrelizumab reduces serum KFLC concentration in MS patients. However, serum KFLC concentration is not able to predict disease activity in these MS patients.

**Supplementary Information:**

The online version contains supplementary material available at 10.1186/s42466-025-00419-7.

## Introduction

Kappa free light chains (KFLC) represent a surrogate marker for activity of B-cell lineage cells and especially plasma cells [1, 2]. Therefore, the level of KFLC in serum are recognized as biomarker for inflammation in various autoimmune diseases, e.g. rheumatoid arthritis or Sjögren´s disease [3–5]. Treatment with rituximab, a B-cell depleting monoclonal antibody against CD20, has been shown to result in a decrease of serum KFLC correlating with reduced disease activity in systemic rheumatoid diseases [6, 7]. Multiple sclerosis (MS) is a chronic inflammatory disease of the central nervous systems (CNS) and therefore the focus has been on the intrathecal production of KFLC [1]. The intrathecal fraction of KFLC has been increasingly recognized as biomarker of central nervous system (CNS) inflammation in MS and other neuro-inflammatory diseases and was therefore proposed for inclusion into the latest proposed revision of the McDonald criteria as additional diagnostic criterion [1, 8]. Since intrathecal immunoglobulin (Ig) synthesis is an important pathophysiological component in the development of inflammatory activity associated with MS, the byproducts of Ig synthesis and secretion such as KFLC in cerebrospinal fluid (CSF) represent a promising biomarker for inflammatory activity [1, 8–12]. Therefore, the influence of the disease progression of MS as well as of disease-modifying therapies (DMT) on CSF concentrations of KFLC has been investigated to establish if intrathecal KFLC-production can serve as biomarker for therapy and disease monitoring. No influence of treatment with interferon-β-1a, fingolimod or alemtuzumab on KFLC concentration in CSF and serum was reported, whereas intrathecal application of rituximab led to a significant increase in KFLC concentration in CSF [13–15]. In contrast, a cross-sectional study reported a decreased intrathecal KFLC fraction in MS patients receiving highly efficient DMT such as alemtuzumab, natalizumab, mitoxantrone and CD20-depleting therapies [16].

In contrast to the abundance of data regarding the intrathecal fraction of KFLC as a promising biomarker for disease activity in MS, data on serum KFLC in MS and the impact of immunomodulatory therapies are scarce. In a previous study, we investigated the influence of different acute treatments on the serum concentration of KFLC and found a rapid decrease in serum KFLC concentration by the intravenous administration of methylprednisolone [17].

Since KFLC in CSF and serum represent products of Ig synthesized by B cells, which are mainly characterized by expression of CD20, they might serve as a biomarker of CD20-depleting therapies such as ocrelizumab [18]. Ocrelizumab is a humanized monoclonal anti-CD20 antibody, which has been approved for the treatment of MS patients with either a relapsing- (RMS) or primary-progressive (PPMS) disease course [19, 20]. Effective and profound depletion of CD20^+^ B and T cells has been demonstrated already two weeks after a single application of 300 mg ocrelizumab [21]. The number of CD20^+^ lymphocytes in the peripheral blood is often analyzed to monitor the efficacy of treatment during therapy with a CD20 antibody. However, the degree of depletion of CD20^+^ lymphocytes has been shown to not convincingly reflect disease activity in MS patients [22, 23]. Therefore, new biomarkers for treatment decisions in patients with MS are needed [24, 25].

The aim of the present study was to assess the impact of treatment with the CD20 antibody ocrelizumab on serum KFLC levels and the ability of serum KFLC to monitor disease activity.

## Methods

### Patients

A total of 50 patients were prospectively included in this monocentric longitudinal study after giving written informed consent (8787_BO_K_2019). All patients presented to the Department of Neurology at Hannover Medical School (MHH) between 2018 and 2021. Patients were diagnosed with either RMS or PPMS and were treated with ocrelizumab. The therapeutic regimen of all patients consisted of an initial intravenous dose of 300 mg ocrelizumab, followed by a second dose of 300 mg after 14 days. Thereafter, a dose of 600 mg ocrelizumab was infused every 6 months. Serum samples of all included patients were collected immediately before the start of ocrelizumab treatment and after two years, when a total ocrelizumab dose of 2400 mg was infused. Previous DMT in the last 12 months prior to start of ocrelizumab was recorded. Patients were considered treatment-naïve if they had not received DMT ever before initiation of ocrelizumab therapy. DMT were discontinued and ocrelizumab was started after remission of therapeutic effects of the DMT. Clinical examinations including Expanded Disability Status Scale (EDSS) and the timed 25-foot walk test (T25FWT) were performed every 6 to 12 months [26, 27].

### Disease activity

Clinical disease activity was defined as the occurrence of one or more relapses in RMS patients, which must have resulted in an increase of at least one point in any functional system score of the EDSS [26]. Additionally, progression independent of relapse activity (PIRA) was also considered as clinical disease activity in RMS and PPMS patients and was evaluated according to previously established criteria [28]. Confirmed disability worsening was determined based on a relapse-dependent or relapse-independent EDSS increase of + 1.5 points from a baseline of 0, + 1.0 point from a baseline between 1 and 5.5, or + 0.5 points from a baseline of ≥ 6.0; this change had to be confirmed after six months [28]. MS disease activity in magnetic resonance imaging (MRI) was defined as onset of new T2 lesions in at least yearly performed 1.5 to 3T MRI in accordance with the MAGNIMS-CMSC-NAIMS guidelines, if they were typical of MS and appeared after treatment initiation [29].

### Analytical procedures

All samples were analyzed according to routine diagnostic procedures in the Neurochemistry Laboratory of the Department of Neurology at MHH. The concentrations of albumin, IgG, IgM and IgA in the serum samples were measured by Kinetic nephelometry (Beckman Coulter IMMAGE, Brea, CA, USA) The following reference values for serum immunoglobulins were used: IgG ≥ 7 g/l, IgA ≥ 0.7 g/l, IgM ≥ 0.4 g/l [30, 31]. Serum free light chains kappa were measured by nephelometry using the N Latex FLC kappa kit (Siemens Healthcare Diagnostics Products GmbH, Erlangen, Germany) according to the manufacturers protocol on the Atellica Neph 630 System (Siemens Healthcare Diagnostics Products GmbH, Erlangen, Germany). The serum pre-dilution was set to 1:100. The lower limit of quantification of the assay was 0.034 mg/l. According to the manufacturer, a reference concentration for serum KFLC ranging from 3.3 mg/l to 19.4 mg/l was considered [31]. 8-color fluorescence-activated cell sorting (FACS) analyses were routinely performed in the Institute of Clinical Chemistry of Hannover Medical School as part of the routine work-up according to international guidelines [32].

### Statistical analysis

Statistical analysis was performed using GraphPad Prism (La Jolla, CA, USA; version 10.1.2). The statistical significance level was set at 5%. The D’Agostino & Pearson omnibus normality test was used to assess the normal distribution of the values. Data were described as minimum, maximum (min-max) and mean unless otherwise stated. The Mann–Whitney U-test was used to analyze independent values. The Kruskal–Wallis test and the Friedman test with Dunn’s Multiple Comparison posthoc test were used for group comparison. Spearman’s r (Gaussian distributed values) and Pearson’s r (nonparametric distributed values) were used to test for correlation and the p-values and correlation coefficient (ρ) were presented.

## Results

### Patient characteristics

Of the included patients, 58% (29/50) were females with a predominance in the RMS group (RMS: 63%, 24/38; PPMS: 42%, 5/12) and a median age of 40 years in RMS and 45.5 in PPMS at initiation of ocrelizumab (Table 1). Most of the RMS patients were already treated before initiation of ocrelizumab (71%, 27/38), with fumaric acids being the most prevalent previous DMT. As shown in Table 1 and supplemental Fig. 1, pathologically elevated serum KFLC concentrations or distinct renal function impairment (below an eGFR of 50 ml/min/1.73 m²) were not observed at any time point and a highly significant correlation between eGFR but not serum creatinine and serum KFLC was observed.

**Table 1:** Demographical and clinical data of multiple sclerosis patients

	Total multiple sclerosis patients (*n* = 50)	Relapsing multiple sclerosis (*n* = 38)	Primary-progressive multiple sclerosis (*n* = 12)
Age in years, median (IQR)	41 (30–50)	40 (28–49)	45.5 (33–54)
Females, n (%)	29 (58%)	24 (63%)	5 (42%)
Time since MS diagnosis [months], median (IQR)	34 (5-136)	29 (4-155)	63 (12–131)
Expanded disability status scale (EDSS) score, median (IQR)	3.5 (2-5.5)	2.5 (1.5-4)	5.5 (1.5-4)
C-reactive protein [mg/l], median (IQR)	0.75 (0.4-2)	0.95 (0.5–2.5)	0.5 (0.3–0.85)
Renal function estimated by eGFR (ml/min/1.73 m²) according to CKD-EPI equation, median (IQR)	100 (90–109)	102 (92–109)	94 (90–114)
Patients with preceding disease-modifying therapies within the last 12 months before initiation of ocrelizumab, n (%)	30 (60%)	27 (71%)	3 (25%)
Preceding disease-modifying therapy with:			
Fumaric acids, n (%)	11/30 (37%)	10/27 (37%)	1/3 (33%)
Glatirameracetate, n (%)	4/30 (13%)	4/27 (15%)	-
Natalizumab, n (%)	4/30 (13%)	4/27 (15%)	-
Fingolimod, n (%)	3/30 (10%)	3/27 (11%)	-
Teriflunomide, n (%)	3/30 (10%)	2/27 (7%)	1/3 (33%)
Interferons, n (%)	2/30 (7%)	2/27 (7%)	-
Daclizumab, n (%)	2/30 (7%)	2/27 (7%)	
Quarterly intravenous mitoxantrone application, n (%)	1/30 (3%)	-	1/3 (33%)

IQR = interquartile range

## The influence of Ocrelizumab treatment on KFLC, Immunoglobulins and leukocyte populations

**Fig. 1:**
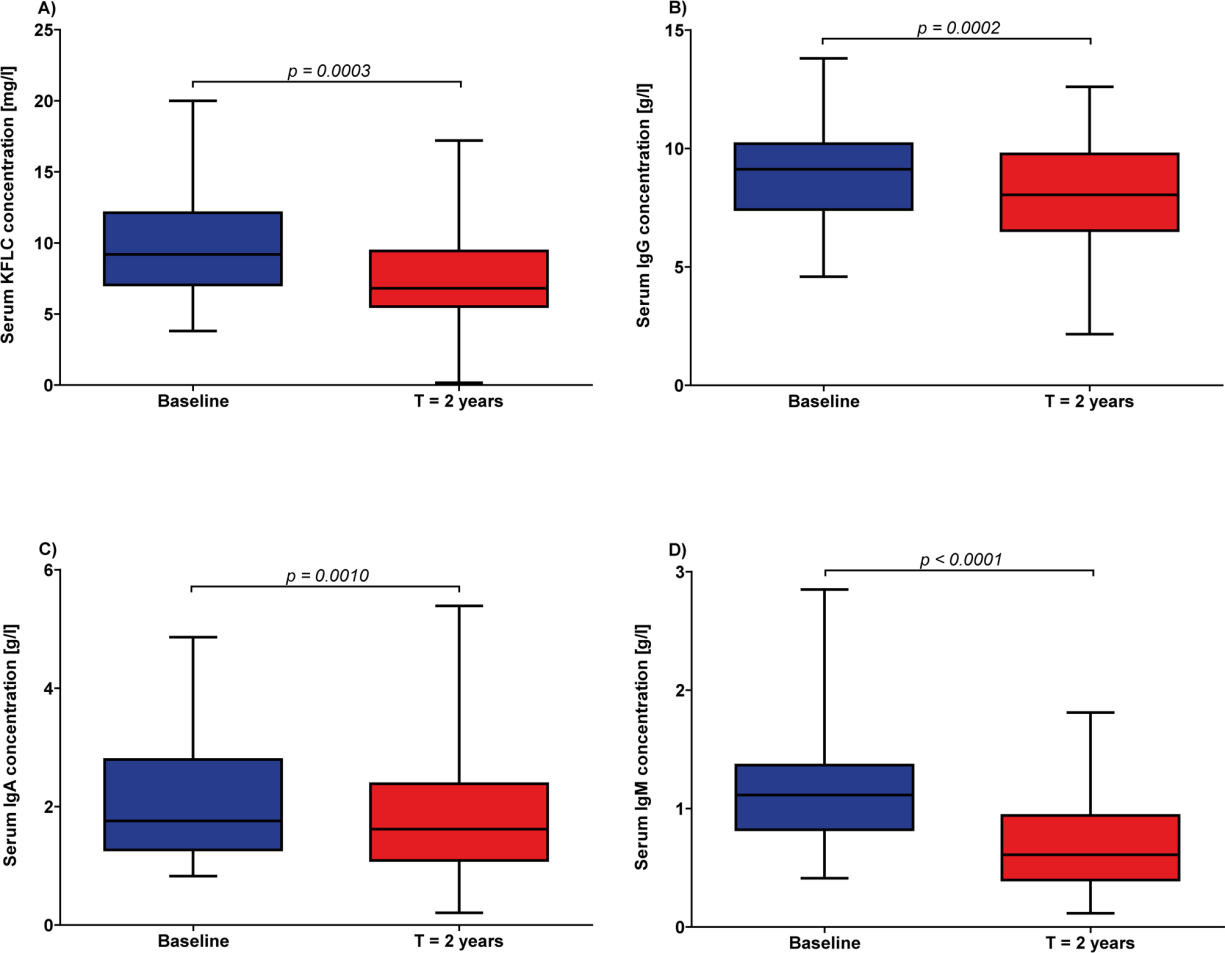
Comparison of kappa free light chain (KFLC) and immunoglobulin (Ig) G, A, and M concentrations at baseline and two years under ocrelizumab treatment. Depicted are KFLC **(A)**, IgG **(B)**, IgA **(C)** and IgM **(D)** concentrations in serum. The level of statistical significance is indicated above the line

Serum KFLC concentration decreased significantly after two years of ocrelizumab treatment compared to baseline concentration from a mean of 9.5 mg/l (± standard deviation (SD) 3.8) to 7.8 mg/l (± 3.8; *p* = 0.0003; Fig. 1, A) with 4% (2/50) of patients presenting with concentration below the reference value of 3.3 mg/l (before treatment no patient was below 3.3 mg/l). Similar to KFLC concentration, concentration of all immunoglobulin classes was significantly lower after two years of ocrelizumab treatment (IgG: 9 g/l (± 2.2) vs. 8 g/l (± 2.2), *p* = 0.0002; IgA: 2 g/l (± 0.9) vs. 1.8 (± 1.0) g/l, *p* = 0.0010; IgM: 1.8 g/l (± 0.5) vs. 0.7 g/l (± 0.4), *p* < 0.0001; Fig. 1, B-D).

Furthermore, the percentage of patients with pathologically low serum Ig concentrations increased significantly after two years of ocrelizumab treatment, reaching statistical significance for IgG and IgM (IgG: 7/50 vs. 18/50, *p* = 0.0198; IgA: 0/50 vs. 4/50, *p* = 0.3839; IgM: 0/50 vs. 13/50, *p* < 0.0001).

While the total number of leukocytes remained unchanged, total lymphocyte count significantly decreased (Table 2). The absolute numbers of neutrophile, eosinophile and basophile granulocytes remained unaffected, whereas the absolute monocyte count increased significantly after two years of ocrelizumab treatment (Table 2). While CD20^+^ and CD19^+^ lymphocyte counts significantly decreased, CD3^+^ lymphocyte counts remained unchanged and CD56^+^ lymphocytes significantly increased (Table 2).

**Table 2:** Flow cytometric and laboratory analysis data of multiple sclerosis (MS) patients

	Baseline, *n* = 50	Two years after treatment onset (after infusion of 2400 mg ocrelizumab), *n* = 50	*p*-value
Leukocytes [absolute count, thousand/µl], mean (SD)	7.0 (2.09)	7.1 (2.52)	0.9662
Lymphocytes [absolute count, thousand/µl], mean (SD)	1.89 (0.83)	1.59 (0.53)	**0.0179**
Neutrophile granulocytes [absolute count, thousand/µl], mean (SD)	4.3 (1.82)	4.7 (2.59)	0.4032
Eosinophile granulocytes [absolute count, thousand/µl], mean (SD)	0.15 (0.12)	0.19 (0.24)	0.3008
Basophile granulocytes [absolute count, thousand/µl], mean (SD)	0.05 (0.03)	0.08 (0.1)	0.0686
Monocytes [absolute count, thousand/µl], mean (SD)	0.56 (0.19)	0.67 (0.21)	**0.0028**
CD20^+^ lymphocytes [absolute count, thousand/µl], mean (SD)	0.19 (0.11)	0.003 (0.015)	**< 0.0001**
CD19^+^ lymphocytes [absolute count, thousand/µl], mean (SD)	0.19 (0.11)	0.003 (0.015)	**< 0.0001**
CD3^+^ lymphocytes [absolute count, thousand/µl], mean (SD)	1.37 (0.45)	1.33 (0.49)	0.8610
CD56^+^ lymphocytes [absolute count, thousand/µl], mean (SD)	0.23 (0.12)	0.27 (0.15)	**0.0194**

SD = standard deviation

### Serum KFLC concentration cannot predict disease activity during ocrelizumab treatment

Relapses and/or PIRA occurred in 22% (11/50) of patients (3 PPMS, 8 RMS) during the 2-year treatment period. 10/11 patients (2 PPMS, 8 RMS) received intravenous corticosteroids of which 7 (3 PPMS, 4 RMS) fully recovered. Overall, the median EDSS score was stable compared with baseline (3.5 and 3.0 respectively, *p* = 0.6068). In five patients, an EDSS score reduction between 0.5 and 1.5 points was observed while EDSS score rose in seven patients (between 0.5 and 2.0 points). Furthermore, 80% (40/50) of patients were able to perform walking tests. A worsening in the T25FWT of at least 20% was found in 14/40 patients when baseline time and time after two years of ocrelizumab treatment were compared (median worsening of 18 s (interquartile range: 14–88 s). MS disease activity in MRI was found in 12% (6/50) of patients with a total of eight new T2 lesions.

Comparison of baseline serum KFLC concentration of patients with and without disease exacerbation with regard to clinical (relapses, PIRA, EDSS worsening) or MRI-morphological (new T2 lesions) disease activity revealed no statistically significant differences (*p* = 0.1724, *p* = 0.1647, respectively). KFLC concentration did not differ significantly in patients with increasing EDSS compared to patients with improved or stable EDSS after two years of ocrelizumab treatment (*p* = 0.7977). For T25FWT, no statistically significant differences concerning serum KFLC concentration between patient samples with and without worsening were found (*p* = 0.5392).

### Serum KFLC correlate with IgG and IgA concentrations

**Fig. 2:**
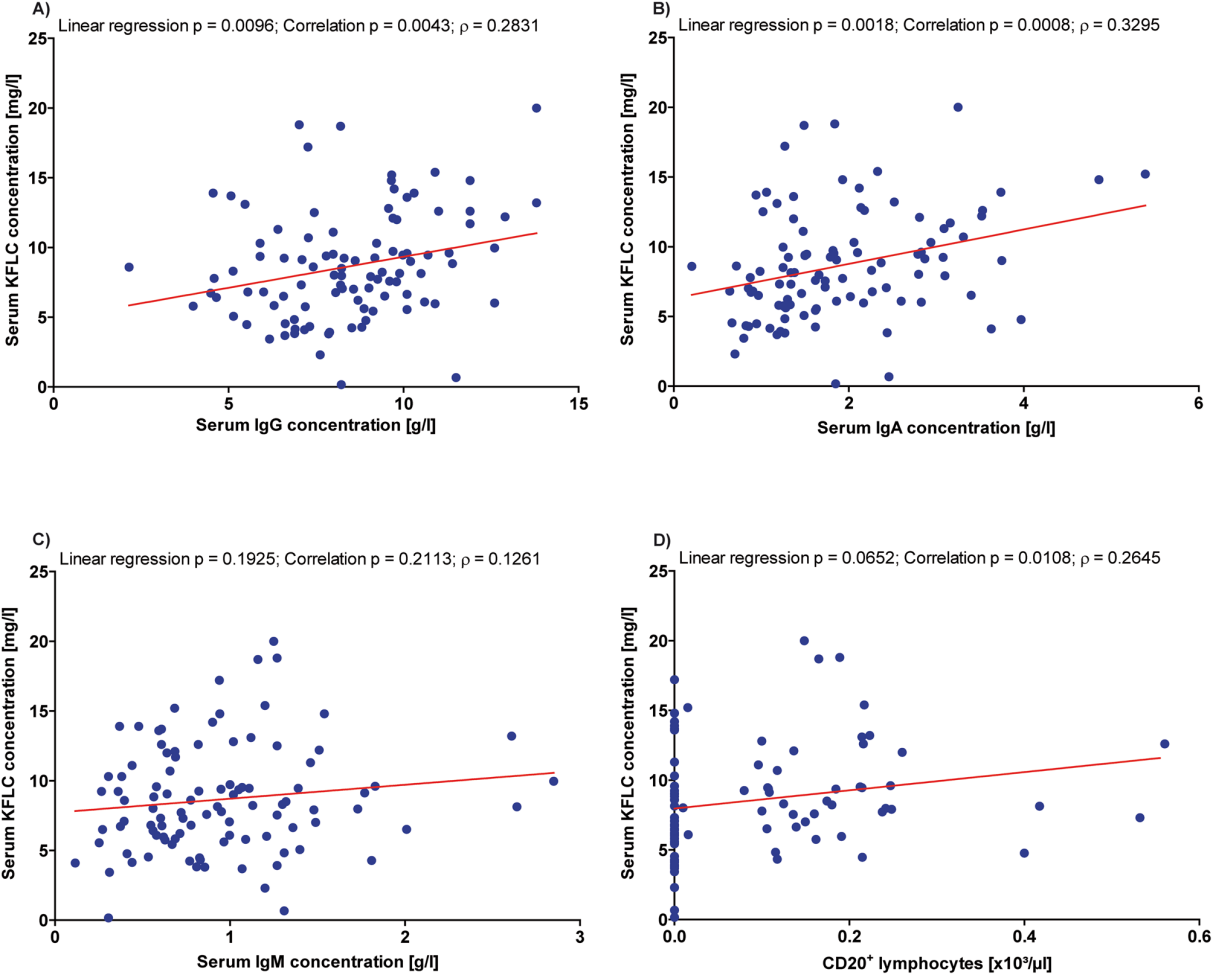
Correlations of kappa free light chain (KFLC) concentration. Depicted are correlations of serum KFLC concentration with immunoglobulin (Ig) G **(A)**, A **(B)** and M **(C)** concentrations and the absolute cell count of CD20^+^ lymphocytes **(D)**. The level of statistical significance of the linear regression and the correlation are given as well as the coefficient of correlation (ρ)

Analysis of serum KFLC concentration at baseline and after two years of ocrelizumab treatment with serum IgG and IgA concentrations revealed a significant correlation and significant linear regression as shown in Fig. 2, A and B. A correlation or significant linear regression did not exist between serum KFLC and IgM concentration (Fig. 2, C). Serum KFLC concentration and the percentage of CD20^+^ lymphocytes showed a significant correlation (Fig. 2D).

## Discussion

Depletion of CD20^+^ lymphocytes with ocrelizumab has been shown to be effective in the treatment of RMS and PPMS patients. Since CD20^+^ T and B cells are targeted by ocrelizumab treatment, markers of B cell activity in serum including KFLC, immunoglobulins, as well as lymphocyte subsets were investigated in the present study. In addition, this is the first study to evaluate serum KFLC concentrations for their potential as a biomarker for treatment monitoring in MS.

Investigation of clinical and imaging parameters for disease activity did not reveal any significant group differences or correlations with the serum KFLC concentration, indicating the missing capability of KFLC to monitor disease activity under ocrelizumab treatment in the present study. Nevertheless, a significant decrease of serum Ig and KFLC concentrations under ocrelizumab treatment over a time period of two years was observed. Since B cells, which are the origin of these proteins, are effectively depleted under ocrelizumab treatment, a reduction was expectable. This finding is also underlined by the positive correlation between serum KFLC concentration and Ig concentrations as well as CD20^+^ lymphocyte count. However, since many data points of the amount of CD20^+^ lymphocytes clustered near zero on the x-axis, the interpretability of this correlation is limited despite being statistically significant. Interestingly, no correlation was found between IgM and KFLC concentrations in serum. Possible explanations for this phenomenon include different originating cell types of IgM antibodies (naïve B cells) and KFLC (long-living plasma cells) and their different affection by anti-CD20-therapies (targeting long-living plasma cells to a lesser proportion), the lower excessive synthesis of KFLC in the formation of the pentamer IgM antibodies, the different half-life time of KFLC (hours) and IgM antibodies (several days) due to the different molecular weight, and lastly the dominance of IgG and IgA in chronic polyclonal immune activation in autoimmune diseases such as MS [1, 2, 16, 17, 33–36]. The combination of these mechanisms could lead to a decoupling of IgM and KFLC concentrations in serum and therefore the observed missing correlation. Contrarily, the cross-sectional study of Süße et al. did not report of significant concentration differences in serum between patients taking high effective DMT, moderately effective DMT and no DMT [16]. A possible explanation might be that the time period under treatment of the included patients in the very high effective DMT cohort was rather short (median 88 days), thus significant differences in serum concentration compared with treatment-naïve patients might not have been found at that early time point [16]. In addition, the high effective DMT cohort did not only include patients under CD20-depleting therapies but also other DMT, which not primarily target B cells [16].

Furthermore, interpreting KFLC concentration, renal function impairment as possible cause for pathologically elevated serum KFLC concentration as well as an increased synthesis of KFLC should be considered [37, 38]. In the present study, pathologically elevated serum KFLC concentrations at baseline as well as distinct renal function impairment were not observed.

Lastly, the role of serum KFLC as possible predictors for disease progression in MS or conversion from clinically isolated syndrome to MS was not investigated yet. In contrast to serum concentrations, the prediction of disease and disability progression based on KFLC concentration in CSF has been extensively studied, yielding conflicting results [2, 39, 40]. Some studies found a significant correlation between high KFLC concentration in CSF and early disability or disability progression, which was mainly estimated by EDSS in patients with RRMS [41–44]. Berek and colleagues reported that a high KFLC index (> 100) at baseline was associated with a shorter time to clinically definite MS and a higher risk of a second clinical relapse within 24 months from baseline [45]. In contrast, other studies have failed to find a correlation between disease progression as measured by EDSS and KFLC concentration in CSF or KFLC indices [14, 46–48]. Reports in the literature are also quite heterogeneous regarding the correlation of KFLC in CSF with MRI parameters of CNS inflammation [2]. Some studies have reported significant correlations between elevated KFLC concentration in CSF or KFLC indices and brain atrophy, brain lesion pattern, or T2-lesion volume in MS patients [45, 49, 50]. In contrast, other studies have not found a significant association between KFLC and brain damage according to MRI or the localization and load of MRI abnormalities [14, 51, 52]. Since this is the first study to investigate the association between MS disease activity and serum KFLC concentrations, no data are yet available to enlighten this issue. In the present study, serum KFLC concentration did not appear to be a suitable biomarker for disease or treatment monitoring, since no correlation with clinical or imaging signs for disease activity was observed. In general, the missing association of serum KFLC with MS disease activity, even under B cell targeting anti-CD20 therapy, might be due to the reflection of a broader, polyclonal, and systemic immune action of serum KFLC, which not necessarily and specifically mirrors the compartmentalized auto-inflammatory processes in the CNS, which are characteristic for MS [1, 2]. This is in contrast to systemic autoimmune diseases such as rheumatoid arthritis or Sjögren´s disease, in which there was a correlation between reduction of KFLC in serum and disease activity under treatment with anti-CD20 therapy with rituximab [6, 7]. Although in our study a higher than expected rate of patients with disease activity of about 20% was observed, the limitation of the low number of included patients, and the inclusion of both, RMS and PPMS patients leading to missing feasibility of subgroup analyses, might be another explanatory factor for the lacking associations between MS disease activity and serum KFLC concentrations. Lastly, the serum samples investigated in our study were not obtained at the time point of the disease activity. For example, none of the samples from patients with relapsing disease activity were gained at the time point of the relapse, but at year two under treatment with ocrelizumab. Therefore, associations between relapsing disease activity in MS and serum KFLC concentrations might be found, when samples are investigated, which were gained at the onset of the new symptoms.

## Conclusion

Infusion of the anti-CD20 monoclonal antibody ocrelizumab resulted in effective depletion of CD20^+^ lymphocytes and a consecutive decrease in serum KFLC levels. The reduced serum KFLC levels were not able to predict disease activity in these MS patients.

## Supplementary Information

Below is the link to the electronic supplementary material.

Supplementary Material 1

Supplementary Material 2

## Data Availability

The datasets used and/or analyzed during the current study are available from the corresponding author on reasonable request.

## References

[1] Hutchison, C. A., Basnayake, K., & Cockwell, P. (2009). Serum free light chain assessment in monoclonal gammopathy and kidney disease. *Nature Reviews Nephrology*, *5*(11), 621–628. 10.1038/nrneph.2009.151 19786994 10.1038/nrneph.2009.151

[2] Konen, F. F., Wurster, U., Schwenkenbecher, P., Gerritzen, A., Groß, C. C., Eichhorn, P., Harrer, A., Isenmann, S., Lewczuk, P., Lewerenz, J., Leypoldt, F., Otto, M., Regeniter, A., Roskos, M., Ruprecht, K., Spreer, A., Strik, H., Uhr, M., Wick, M., Wildemann, B., Wiltfang, J., Zimmermann, T., Hannich, M., Khalil, M., Tumani, H., Süße, M., Skripuletz, T., & German Society for Cerebrospinal Fluid Diagnostics and Clinical Neurochemistry (DGLN e.V.). (2025). Oligoclonal bands and kappa free light chains: Competing parameters or complementary biomarkers? *Autoimmunity Reviews*, *24*(5), 103765. 10.1016/j.autrev.2025.103765 39947571 10.1016/j.autrev.2025.103765

[3] Gottenberg, J. E., Aucouturier, F., Goetz, J., Sordet, C., Jahn, I., Busson, M., Cayuela, J. M., Sibilia, J., & Mariette, X. (2007). Serum Immunoglobulin free light chain assessment in rheumatoid arthritis and primary sjogren’s syndrome. *Annals of the Rheumatic Diseases*, *66*(1), 23–27. 10.1136/ard.2006.052159 16569685 10.1136/ard.2006.052159 PMC1798389

[4] Ye, Y., Li, S. L., Xie, M., Jiang, P., Liu, K. G., & Li, Y. J. (2013). Judging disease activity in rheumatoid arthritis by serum free kappa and lambda light chain levels. *The Kaohsiung Journal of Medical Sciences*, *29*(10), 547–553. 10.1016/j.kjms.2013.01.013 24099109 10.1016/j.kjms.2013.01.013 PMC11916879

[5] Kaplan, B., Livneh, A., & Sela, B. A. (2011). Immunoglobulin free light chain dimers in human diseases. *TheScientificWorldJournal*, *11*, 726–735. 10.1100/tsw.2011.65 21442150 10.1100/tsw.2011.65 PMC5720091

[6] Kormelink, T. G., Tekstra, J., Thurlings, R. M., Boumans, M. H., Vos, K., Tak, P. P., Bijlsma, J. W., Lafeber, F. P., Redegeld, F. A., & van Roon, J. A. (2010). Decrease in Immunoglobulin free light chains in patients with rheumatoid arthritis upon rituximab (anti-CD20) treatment correlates with decrease in disease activity. *Annals of the Rheumatic Diseases*, *69*(12), 2137–2144. 10.1136/ard.2009.126441 20679475 10.1136/ard.2009.126441

[7] Verstappen, G. M., Moerman, R. V., van Nimwegen, J. F., van Ginkel, M. S., Bijzet, J., Mossel, E., Vissink, A., Hazenberg, B. P. C., Arends, S., Kroese, F. G. M., & Bootsma, H. (2018). Serum Immunoglobulin free light chains are sensitive biomarkers for monitoring disease activity and treatment response in primary sjögren’s syndrome. *Rheumatology (Oxford England)*, *57*(10), 1812–1821. 10.1093/rheumatology/key180 29982712 10.1093/rheumatology/key180

[8] Montalban, X. (2024). 2024 revisions of the McDonald criteria. ECTRIMS: 40th Congress of the European Committee for Treatment and Research in Multiple Sclerosis, 18–20 September 2024. https://ectrims.eu/mcdonald-diagnostic-criteria. Accessed 5 November 2024.

[9] Cencioni, M. T., Mattoscio, M., Magliozzi, R., Bar-Or, A., & Muraro, P. A. (2021). B cells in multiple sclerosis - from targeted depletion to immune reconstitution therapies. *Nature Reviews Neurology*, *17*(7), 399–414. 10.1038/s41582-021-00498-5 34075251 10.1038/s41582-021-00498-5

[10] Süße, M., Hannich, M., Petersmann, A., Zylla, S., Pietzner, M., Nauck, M., & Dressel, A. (2018). Kappa free light chains in cerebrospinal fluid to identify patients with oligoclonal bands. *European Journal of Neurology*, *25*(9), 1134–1139. 10.1111/ene.13667 29683546 10.1111/ene.13667

[11] Nakano, T., Matsui, M., Inoue, I., Awata, T., Katayama, S., & Murakoshi, T. (2011). Free Immunoglobulin light chain: Its biology and implications in diseases. *Clinica Chimica Acta; International Journal of Clinical Chemistry*, *412*(11–12), 843–849. 10.1016/j.cca.2011.03.007 21396928 10.1016/j.cca.2011.03.007

[12] Schwenkenbecher, P., Konen, F. F., Wurster, U., Witte, T., Gingele, S., Sühs, K. W., Stangel, M., & Skripuletz, T. (2019). Reiber’s diagram for kappa free light chains: The new standard for assessing intrathecal synthesis?? *Diagnostics (Basel Switzerland)*, *9*(4), 194. 10.3390/diagnostics9040194 31744096 10.3390/diagnostics9040194 PMC6963502

[13] Topping, J., Dobson, R., Lapin, S., Maslyanskiy, A., Kropshofer, H., Leppert, D., Giovannoni, G., & Evdoshenko, E. (2016). The effects of intrathecal rituximab on biomarkers in multiple sclerosis. *Multiple Sclerosis and Related Disorders*, *6*, 49–53. 10.1016/j.msard.2016.01.001 27063622 10.1016/j.msard.2016.01.001

[14] Rosenstein, I., Rasch, S., Axelsson, M., Novakova, L., Blennow, K., Zetterberg, H., & Lycke, J. (2021). Kappa free light chain index as a diagnostic biomarker in multiple sclerosis: A real-world investigation. *Journal of Neurochemistry*, *159*(3), 618–628. 10.1111/jnc.15500 34478561 10.1111/jnc.15500

[15] Rudick, R. A., Cookfair, D. L., Simonian, N. A., Ransohoff, R. M., Richert, J. R.,Jacobs, L. D., Herndon, R. M., Salazar, A. M., Fischer, J. S., Granger, C. V., Goodkin,D. E., Simon, J. H., Bartoszak, D. M., Bourdette, D. N., Braiman, J., Brownscheidle,C. M., Coats, M. E., Cohan, S. L., Dougherty, D. S., Kinkel, R. P., … Whitham, R.H. (1999). Cerebrospinal fluid abnormalities in a phase III trial of Avonex (IFNbeta-1a)for relapsing multiple sclerosis. The Multiple Sclerosis Collaborative Research Group.*Journal of neuroimmunology*, *93*(1–2), 8–14. 10.1016/s0165-5728(98)00174-x. 10.1016/s0165-5728(98)00174-x 10378864

[16] Süße, M., Konen, F. F., Schwenkenbecher, P., Budde, K., Nauck, M., Grothe, M., Hannich, M. J., & Skripuletz, T. (2022). Decreased intrathecal concentrations of free light chains kappa in multiple sclerosis patients taking very high effective Disease-Modifying treatment. *Diagnostics (Basel Switzerland)*, *12*(3), 720. 10.3390/diagnostics12030720 35328273 10.3390/diagnostics12030720 PMC8947149

[17] Konen, F. F., Wurster, U., Witte, T., Jendretzky, K. F., Gingele, S., Tumani, H., Sühs, K. W., Stangel, M., Schwenkenbecher, P., & Skripuletz, T. (2020). The impact of Immunomodulatory treatment on kappa free light chains as biomarker in neuroinflammation. *Cells*, *9*(4), 842. 10.3390/cells9040842 32244362 10.3390/cells9040842 PMC7226742

[18] Vecchio, D., Bellomo, G., Serino, R., Virgilio, E., Lamonaca, M., Dianzani, U., Cantello, R., Comi, C., & Crespi, I. (2020). Intrathecal kappa free light chains as markers for multiple sclerosis. *Scientific Reports*, *10*(1), 20329. 10.1038/s41598-020-77029-7 33230241 10.1038/s41598-020-77029-7 PMC7683527

[19] Hauser, S. L., Bar-Or, A., Comi, G., Giovannoni, G., Hartung, H. P., Hemmer, B.,Lublin, F., Montalban, X., Rammohan, K. W., Selmaj, K., Traboulsee, A., Wolinsky,J. S., Arnold, D. L., Klingelschmitt, G., Masterman, D., Fontoura, P., Belachew, S.,Chin, P., Mairon, N., Garren, H., … OPERA I and OPERA II Clinical Investigators (2017).Ocrelizumab versus Interferon Beta-1a in Relapsing Multiple Sclerosis. *The New England journal of medicine*, *376*(3), 221–234. 10.1056/NEJMoa1601277. 10.1056/NEJMoa1601277 28002679

[20] Montalban, X., Hauser, S. L., Kappos, L., Arnold, D. L., Bar-Or, A., Comi, G., de Seze, J., Giovannoni, G., Hartung, H. P., Hemmer, B., Lublin, F., Rammohan, K. W., Selmaj, K., Traboulsee, A., Sauter, A., Masterman, D., Fontoura, P., Belachew, S., Garren, H., Mairon, N., & ORATORIO Clinical Investigators. (2017). Ocrelizumab versus placebo in primary progressive multiple sclerosis. *The New England Journal of Medicine*, *376*(3), 209–220. 10.1056/NEJMoa1606468 28002688 10.1056/NEJMoa1606468

[21] Gingele, S., Jacobus, T. L., Konen, F. F., Hümmert, M. W., Sühs, K. W., Schwenkenbecher, P., Ahlbrecht, J., Möhn, N., Müschen, L. H., Bönig, L., Alvermann, S., Schmidt, R. E., Stangel, M., Jacobs, R., & Skripuletz, T. (2018). Ocrelizumab depletes CD20⁺ T cells in multiple sclerosis patients. *Cells*, *8*(1), 12. 10.3390/cells8010012 30597851 10.3390/cells8010012 PMC6356421

[22] Freeman, S. A., Lemarchant, B., Alberto, T., Boucher, J., Outteryck, O., Labalette, M., Rogeau, S., Dubucquoi, S., & Zéphir, H. (2023). Assessing sustained B-Cell depletion and disease activity in a French multiple sclerosis cohort treated by Long-Term IV Anti-CD20 antibody therapy. *Neurotherapeutics: the Journal of the American Society for Experimental NeuroTherapeutics*, *20*(6), 1707–1722. 10.1007/s13311-023-01446-5 37882961 10.1007/s13311-023-01446-5 PMC10684468

[23] Baker, D., MacDougall, A., Kang, A. S., Schmierer, K., Giovannoni, G., & Dobson, R. (2022). CD19 B cell repopulation after ocrelizumab, alemtuzumab and cladribine: Implications for SARS-CoV-2 vaccinations in multiple sclerosis. *Multiple Sclerosis and Related Disorders*, *57*, 103448. 10.1016/j.msard.2021.103448 34902760 10.1016/j.msard.2021.103448 PMC8642825

[24] Mrochen, A., Meuth, S. G., & Pfeuffer, S. (2025). Should we stay or should we go? Recent insights on drug discontinuation in multiple sclerosis. *Neurological Research and Practice*, *7*(1), 25. 10.1186/s42466-025-00379-y 40254626 10.1186/s42466-025-00379-y PMC12010584

[25] Bayas, A., Mansmann, U., Ön, B. I., Hoffmann, V. S., Berthele, A., Mühlau, M., Kowarik,M. C., Krumbholz, M., Senel, M., Steuerwald, V., Naumann, M., Hartberger, J., Kerschensteiner,M., Oswald, E., Ruschil, C., Ziemann, U., Tumani, H., Vardakas, I., Albashiti, F.,Kramer, F., … ProVal-MS study group (2024). Prospective study validating a multidimensional treatment decision score predicting the 24-month outcome in untreated patients with clinically isolated syndrome and early relapsing-remitting multiple sclerosis, the ProVal-MS study. *Neurological research and practice*, *6*(1), 15. 10.1186/s42466-024-00310-x. 10.1186/s42466-024-00310-x PMC10918966 38449051

[26] Kurtzke, J. F. (1983). Rating neurologic impairment in multiple sclerosis: An expanded disability status scale (EDSS). *Neurology*, *33*(11), 1444–1452. 10.1212/wnl.33.11.1444 6685237 10.1212/wnl.33.11.1444

[27] Kaufman, M., Moyer, D., & Norton, J. (2000). The significant change for the timed 25-foot walk in the multiple sclerosis functional composite. *Multiple Sclerosis (Houndmills Basingstoke England)*, *6*(4), 286–290. 10.1177/135245850000600411 10962550 10.1177/135245850000600411

[28] Kappos, L., Wolinsky, J. S., Giovannoni, G., Arnold, D. L., Wang, Q., Bernasconi, C., Model, F., Koendgen, H., Manfrini, M., Belachew, S., & Hauser, S. L. (2020). Contribution of Relapse-Independent progression vs Relapse-Associated worsening to overall confirmed disability accumulation in typical relapsing multiple sclerosis in a pooled analysis of 2 randomized clinical trials. *JAMA Neurology*, *77*(9), 1132–1140. 10.1001/jamaneurol.2020.1568 32511687 10.1001/jamaneurol.2020.1568 PMC7281382

[29] Wattjes, M. P., Ciccarelli, O., Reich, D. S., Banwell, B., de Stefano, N., Enzinger, C., Fazekas, F., Filippi, M., Frederiksen, J., Gasperini, C., Hacohen, Y., Kappos, L., Li, D. K. B., Mankad, K., Montalban, X., Newsome, S. D., Oh, J., Palace, J., Rocca, M. A., Sastre-Garriga, J., & North American Imaging in Multiple Sclerosis Cooperative MRI guidelines working group. (2021). 2021 MAGNIMS-CMSC-NAIMS consensus recommendations on the use of MRI in patients with multiple sclerosis. *The Lancet Neurology*, *20*(8), 653–670. 10.1016/S1474-4422(21)00095-8 34139157 10.1016/S1474-4422(21)00095-8

[30] Dati, F., Schumann, G., Thomas, L., Aguzzi, F., Baudner, S., Bienvenu, J., Blaabjerg, O., Blirup-Jensen, S., Carlström, A., Petersen, P. H., Johnson, A. M., Milford-Ward, A., Ritchie, R. F., Svendsen, P. J., & Whicher, J. (1996). Consensus of a group of professional societies and diagnostic companies on guidelines for interim reference ranges for 14 proteins in serum based on the standardization against the IFCC/BCR/CAP Reference Material (CRM 470). International Federation of Clinical Chemistry. Community Bureau of Reference of the Commission of the European Communities. College of American Pathologists. *European journal of clinical chemistry and clinical biochemistry: journal of the Forum of European Clinical Chemistry Societies*, *34*(6), 517–520. 8831057

[31] Katzmann, J. A., Clark, R. J., Abraham, R. S., Bryant, S., Lymp, J. F., Bradwell, A. R., & Kyle, R. A. (2002). Serum reference intervals and diagnostic ranges for free kappa and free lambda Immunoglobulin light chains: Relative sensitivity for detection of monoclonal light chains. *Clinical Chemistry*, *48*(9), 1437–1444. 12194920

[32] Cossarizza, A., Chang, H. D., Radbruch, A., Abrignani, S., Addo, R., Akdis, M., Andrä,I., Andreata, F., Annunziato, F., Arranz, E., Bacher, P., Bari, S., Barnaba, V., Barros-Martins,J., Baumjohann, D., Beccaria, C. G., Bernardo, D., Boardman, D. A., Borger, J., Böttcher,C., … Yang, J. (2021). Guidelines for the use of flow cytometry and cell sorting in immunological studies (third edition). *European journal of immunology*, *51*(12), 2708–3145. 10.1002/eji.202170126. 10.1002/eji.202170126 PMC11115438 34910301

[33] Das, M., Mead, G. P., Sreekanth, V., Anderson, J., Blair, S., Howe, T., Cavet, J., & Liakopoulou, E. (2005). Serum free light chain (SFLC) concentration kinetics in patients receiving bortezomib: Temporary Inhibition of protein synthesis and early biomarker for disease response. *Blood*, *106*(11), 5094. 10.1182/blood.V106.11.5094.5094

[34] Mead, G. P., Reid, S. D., Augustson, B. M., Drayson, M. T., Bradwell, A. R., & Child, J. A. (2004). Correlation of serum free light chains and bone marrow plasma cell infiltration in multiple myeloma. *Blood*, *104*(11), 4865. 10.1182/blood.V104.11.4865.4865

[35] Robinson, W. H., Fiorentino, D., Chung, L., Moreland, L. W., Deodhar, M., Harler, M. B., Saulsbery, C., & Kunder, R. (2024). Cutting-edge approaches to B-cell depletion in autoimmune diseases. *Frontiers in Immunology*, *15*, 1454747. 10.3389/fimmu.2024.1454747 39445025 10.3389/fimmu.2024.1454747 PMC11497632

[36] Willison, A. G., Hagler, R., Weise, M., Elben, S., Huntemann, N., Masanneck, L.,Pfeuffer, S., Lichtenberg, S., Golombeck, K. S., Preuth, L. M., Rolfes, L., Öztürk,M., Ruck, T., Melzer, N., Korsen, M., Hauser, S. L., Hartung, H. P., Lang, P. A.,Pawlitzki, M., Räuber, S., … Meuth, S. G. (2025). Effects of Anti-CD20 Antibody Therapy on Immune Cell Dynamics in Relapsing-Remitting Multiple Sclerosis. *Cells*, *14*(7), 552. 10.3390/cells14070552. 10.3390/cells14070552 PMC11988809 40214505

[37] Konen, F. F., Schwenkenbecher, P., Wurster, U., Jendretzky, K. F., Möhn, N., Gingele, S., Sühs, K. W., Hannich, M. J., Grothe, M., Witte, T., Stangel, M., Süße, M., & Skripuletz, T. (2021). The influence of renal function impairment on kappa free light chains in cerebrospinal fluid. *Journal of Central Nervous System Disease*, *13*, 11795735211042166. 10.1177/11795735211042166 34840504 10.1177/11795735211042166 PMC8619759

[38] Hannich, M. J., Konen, F. F., Gag, K., Alkhayer, A., Türker, S. N., Budde, K., Nauck, M., Wurster, U., Dressel, A., Skripuletz, T., & Süße, M. (2024). Implications of monoclonal gammopathy and isoelectric focusing pattern 5 on the free light chain kappa diagnostics in cerebrospinal fluid. *Clinical Chemistry and Laboratory Medicine*, *63*(1), 147–153. 10.1515/cclm-2023-1468 39039726 10.1515/cclm-2023-1468

[39] Tumani, H., Tourtellotte, W. W., Peter, J. B., & Felgenhauer, K. (1998). Acute optic neuritis: Combined immunological markers and magnetic resonance imaging predict subsequent development of multiple sclerosis. The optic neuritis study group. *Journal of the Neurological Sciences*, *155*(1), 44–49. 10.1016/s0022-510x(97)00272-4 9562321 10.1016/s0022-510x(97)00272-4

[40] Klein, A., Selter, R. C., Hapfelmeier, A., Berthele, A., Müller-Myhsok, B., Pongratz, V., Gasperi, C., Zimmer, C., Mühlau, M., & Hemmer, B. (2019). CSF parameters associated with early MRI activity in patients with MS. *Neurology(R) Neuroimmunology & Neuroinflammation*, *6*(4), e573. 10.1212/NXI.0000000000000573 31355309 10.1212/NXI.0000000000000573 PMC6624100

[41] Makshakov, G., Nazarov, V., Kochetova, O., Surkova, E., Lapin, S., & Evdoshenko, E. (2015). Diagnostic and prognostic value of the cerebrospinal fluid concentration of Immunoglobulin free light chains in clinically isolated syndrome with conversion to multiple sclerosis. *PloS One*, *10*(11), e0143375. 10.1371/journal.pone.0143375 26606531 10.1371/journal.pone.0143375 PMC4659555

[42] Rudick, R. A., Medendorp, S. V., Namey, M., Boyle, S., & Fischer, J. (1995). Multiple sclerosis progression in a natural history study: Predictive value of cerebrospinal fluid free kappa light chains. *Multiple Sclerosis (Houndmills Basingstoke England)*, *1*(3), 150–155. 10.1177/135245859500100303 9345445 10.1177/135245859500100303

[43] Vecchio, D., Crespi, I., Virgilio, E., Naldi, P., Campisi, M. P., Serino, R., Dianzani, U., Bellomo, G., Cantello, R., & Comi, C. (2019). Kappa free light chains could predict early disease course in multiple sclerosis. *Multiple Sclerosis and Related Disorders*, *30*, 81–84. 10.1016/j.msard.2019.02.001 30738877 10.1016/j.msard.2019.02.001

[44] Salavisa, M., Paixão, P., Ladeira, A. F., Mendes, A., Correia, A. S., Viana, J. F., & Viana-Baptista, M. (2020). Prognostic value of kappa free light chains determination in first-ever multiple sclerosis relapse. *Journal of Neuroimmunology*, *347*, 577355. 10.1016/j.jneuroim.2020.577355 32795735 10.1016/j.jneuroim.2020.577355

[45] Berek, K., Bsteh, G., Auer, M., Di Pauli, F., Grams, A., Milosavljevic, D., Poskaite, P., Schnabl, C., Wurth, S., Zinganell, A., Berger, T., Walde, J., Deisenhammer, F., & Hegen, H. (2021). Kappa-Free light chains in CSF predict early multiple sclerosis disease activity. *Neurology(R) Neuroimmunology & Neuroinflammation*, *8*(4), e1005. 10.1212/NXI.0000000000001005 34049994 10.1212/NXI.0000000000001005 PMC8168046

[46] Presslauer, S., Milosavljevic, D., Brücke, T., Bayer, P., & Hübl, W. (2008). Elevated levels of kappa free light chains in CSF support the diagnosis of multiple sclerosis. *Journal of Neurology*, *255*(10), 1508–1514. 10.1007/s00415-008-0954-z 18685917 10.1007/s00415-008-0954-z

[47] Duranti, F., Pieri, M., Centonze, D., Buttari, F., Bernardini, S., & Dessi, M. (2013). Determination of κFLC and κ index in cerebrospinal fluid: A valid alternative to assess intrathecal Immunoglobulin synthesis. *Journal of Neuroimmunology*, *263*(1–2), 116–120. 10.1016/j.jneuroim.2013.07.006 23916392 10.1016/j.jneuroim.2013.07.006

[48] Desplat-Jégo, S., Feuillet, L., Pelletier, J., Bernard, D., Chérif, A. A., & Boucraut, J. (2005). Quantification of Immunoglobulin free light chains in cerebrospinal fluid by nephelometry. *Journal of Clinical Immunology*, *25*(4), 338–345. 10.1007/s10875-005-5371-9 16133990 10.1007/s10875-005-5371-9

[49] Gaetani, L., Di Carlo, M., Brachelente, G., Valletta, F., Eusebi, P., Mancini, A., Gentili, L., Borrelli, A., Calabresi, P., Sarchielli, P., Ferri, C., Villa, A., & Di Filippo, M. (2020). Cerebrospinal fluid free light chains compared to oligoclonal bands as biomarkers in multiple sclerosis. *Journal of Neuroimmunology*, *339*, 577108. 10.1016/j.jneuroim.2019.577108 31743879 10.1016/j.jneuroim.2019.577108

[50] Nazarov, V., Makshakov, G., Kalinin, I., Lapin, S., Surkova, E., Mikhailova, L., Gilburd, B., Skoromets, A., & Evdoshenko, E. (2018). Concentrations of Immunoglobulin free light chains in cerebrospinal fluid predict increased level of brain atrophy in multiple sclerosis. *Immunologic Research*, *66*(6), 761–767. 10.1007/s12026-018-9058-8 30635824 10.1007/s12026-018-9058-8

[51] Puthenparampil, M., Altinier, S., Stropparo, E., Zywicki, S., Poggiali, D., Cazzola, C., Toffanin, E., Ruggero, S., Grassivaro, F., Zaninotto, M., Plebani, M., & Gallo, P. (2018). Intrathecal K free light chain synthesis in multiple sclerosis at clinical onset associates with local IgG production and improves the diagnostic value of cerebrospinal fluid examination. *Multiple Sclerosis and Related Disorders*, *25*, 241–245. 10.1016/j.msard.2018.08.002 30130707 10.1016/j.msard.2018.08.002

[52] Hassan-Smith, G., Durant, L., Tsentemeidou, A., Assi, L. K., Faint, J. M., Kalra, S., Douglas, M. R., & Curnow, S. J. (2014). High sensitivity and specificity of elevated cerebrospinal fluid kappa free light chains in suspected multiple sclerosis. *Journal of Neuroimmunology*, *276*(1–2), 175–179. 10.1016/j.jneuroim.2014.08.003 25146968 10.1016/j.jneuroim.2014.08.003

