# Supplementary material for: Kappa free light chain concentration in serum is reduced after CD20-depletion with ocrelizumab
Source: Neurol Res Pract. 2025 Aug 22;7(1):58. doi: 10.1186/s42466-025-00419-7 (PMC12372195; doi:10.1186/s42466-025-00419-7)
Supplement: Supplementary file 1 — Supplementary Material 1 [file 42466_2025_419_MOESM1_ESM.docx]

**Supplemental Figure 1.** Correlation of serum concentrations of kappa free light chains (KFLC) with markers of renal function.

Renal function estimated by eGFR (ml/min/1.73 m²) according to CKD-EPI equation and creatinine concentrations from baseline and after 24 months of ocrelizumab treatment.
