# Supplementary figures and images for: Kappa free light chain concentration in serum is reduced after CD20-depletion with ocrelizumab
Source: Neurol Res Pract. 2025 Aug 22;7(1):58. doi: 10.1186/s42466-025-00419-7 (PMC12372195; doi:10.1186/s42466-025-00419-7)

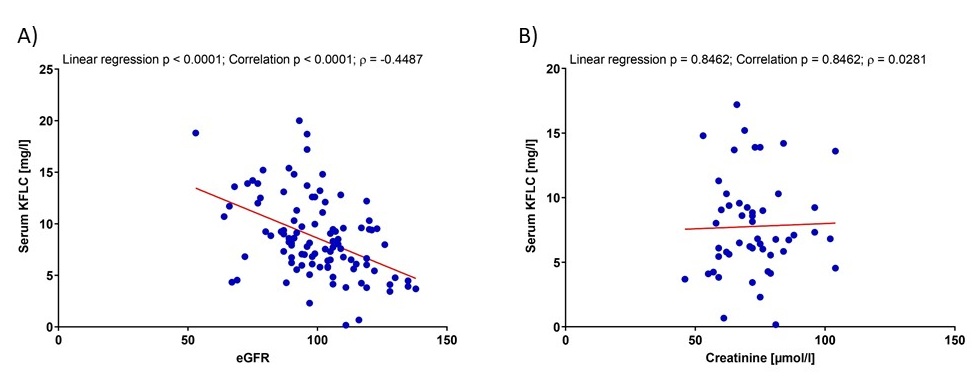

Supplement: Supplementary file 2 — Supplementary Material 2 [file 42466_2025_419_MOESM2_ESM.jpg]
